# Transgenic Cotton Plants Expressing the *HaHR3* Gene Conferred Enhanced Resistance to *Helicoverpa armigera* and Improved Cotton Yield

**DOI:** 10.3390/ijms18091874

**Published:** 2017-08-30

**Authors:** Qiang Han, Zhenzhen Wang, Yunxin He, Yehui Xiong, Shun Lv, Shupeng Li, Zhigang Zhang, Dewen Qiu, Hongmei Zeng

**Affiliations:** 1 The State Key Laboratory for Biology of Plant Diseases and Insect Pests, Institute of Plant Protection, Chinese Academy of Agricultural Sciences, Beijing 100081, China; hanqiang103@gmail.com (Q.H.); wangzhen1567@gmail.com (Z.W.); yehuixiong@mail.tsinghua.edu.cn (Y.X.); lvshun2017@gmail.com (S.L.); shupengli123@gmail.com (S.L.); qiudewen@caas.cn (D.Q.); 2 Cotton Science Research Institute of Hunan Province, Changde 415101, Hunan, China; heyunxin2017@gmail.com (Y.H.); zhangzhigang2018@gmail.com (Z.Z.)

**Keywords:** RNA interference, plant mediated-RNAi, *Helicoverpa armigera*, *HaHR3*, transgenic cotton plants

## Abstract

RNA interference (RNAi) has been developed as an efficient technology. RNAi insect-resistant transgenic plants expressing double-stranded RNA (dsRNA) that is ingested into insects to silence target genes can affect the viability of these pests or even lead to their death. *HaHR3*, a molt-regulating transcription factor gene, was previously selected as a target expressed in bacteria and tobacco plants to control *Helicoverpa armigera* by RNAi technology. In this work, we selected the dsRNA-*HaHR3* fragment to silence *HaHR3* in cotton bollworm for plant mediated-RNAi research. A total of 19 transgenic cotton lines expressing *HaHR3* were successfully cultivated, and seven generated lines were used to perform feeding bioassays. Transgenic cotton plants expressing ds*HaHR3* were shown to induce high larval mortality and deformities of pupation and adult eclosion when used to feed the newly hatched larvae, and 3rd and 5th instar larvae of *H. armigera*. Moreover, *HaHR3* transgenic cotton also demonstrated an improved cotton yield when compared with controls.

## 1. Introduction

Cotton is an important crop worldwide that is mainly used as a textile fiber [1], although its seeds can be processed into cottonseed protein, oil, meals, and industrial raw materials [2,3]. The cotton bollworm (*Helicoverpa armigera*) is one of the major herbivorous insect pests, which causes significant yield losses in cotton, corn, and other crops.

In 1998, lengths of double-stranded RNA (dsRNA) were discovered to silence target genes, triggering an RNA interference (RNAi) response [4]. Since then, RNAi has gradually been developed as an efficient pest control technology that does not leave residual pesticide in the environment [5].

Traditionally, *H. armigera* was controlled by insecticides, although these have adverse effects on human health and surface waters [6]. *Bacillus thuringiensis* (Bt) cotton can effectively kill cotton bollworm, and has shown spectacular success in field trials [7,8]; however, this advantage diminished when many insect pests, such as *H. armigera*, evolved resistance to Bt toxin proteins [9]. Moreover, the cumulative number of major pest species with field-evolved resistance to Bt crops increased from one in 2005 to five in 2010 [10], and Bt proteins cause allergic reactions in some consumers [11]. RNAi insect-resistant transgenic plants express dsRNA that is ingested into target insects and that can affect the viability of these pests or lead to their death [12].

The concept of RNAi plant-mediated pest control was demonstrated by two studies in 2007. Mao et al. [13] silenced a cytochrome P450 gene from cotton bollworm by plant-mediated RNAi, reducing larval tolerance of gossypol. In another study, Baum et al. [14] fed western corn rootworm (WCR) transgenic corn plants expressing WCR dsRNAs that resulted in larval stunting and mortality. These plants showed a significant reduction in WCR feeding damage in a growth chamber assay. Since then, the technology of transgenic plants expressing dsRNA has led to a flourishing new field of research that has been used to control several insect species from various orders. Hundreds of research papers about the combination topics “RNAi”, “plant”, and “insect” can be searched for on the publication database “Web of Science” from Thomson Reuters (August 2015) [15]. Thus, the gene silencing triggered by dsRNA is likely to be adopted in the next generation of insect-resistant transgenic plant breeding.

Molting and metamorphosis are the two most dramatic events in an insect’s lifestyle, and are controlled by two primary hormones: steroid hormone 20-hydroxyecdysone (20-E) and juvenile hormone [16,17,18]. The balance between the two hormones determines insect growth and development. 20-E binds to the ecdysone receptor and the ultraspiracle protein heterodimer to initiate a molting cascade that triggers the expression of transcription factors involved in the onset of metamorphosis [18,19,20]. Hormone receptor 3 (HR3) is a molt-regulating transcription factor that regulates the expression of tissue-specific genes involved in insect molting and metamorphosis, and HR3 orthologs have also been identified in several different insect species [21]. *HaHR3*, which shares a high identity with *HHR3* [22], was cloned from *H. armigera* in a previous study [23]. Early work using *HaHR3* as a target gene to control *H. armigera* by RNAi technology showed that the 5′ terminal 450 bp of dsRNA-*HaHR3* expressed in transgenic tobacco plants was effective at inducing developmental deformity and larval lethality of *H. armigera* [24].

In the present study, we selected pCAM-RNAi-*HaHR3* (the 5′ terminal 450 bp of dsRNA-*HaHR3*) for plant mediated-RNAi research. Transgenic cotton plants expressing ds*HaHR3* were used to feed the newly hatched, and the 3rd and 5th instar larvae of *H. armigera*. Our results demonstrate that transgenic cotton plants can induce high larval mortality and deformity of pupation and adult eclosion.

## 2. Results

### 2.1. Generation of Transgenic Cotton Plants Expressing dsHaHR3

After transforming pCAMBIA-RNAi-*HaHR3* into cotton plants via the pollen tube pathway by injecting 30,000 flowers, plants were grown for 4 months and 80,000 seeds of T_0_ transgenic cotton plants were obtained and sown at the breeding base. A total of 19 generated cotton lines (lines 1 and 2–19) resistant to kanamycin were obtained by four applications of selection. T_1_ and T_2_-generation seeds were then successfully harvested.

**Figure 1:**
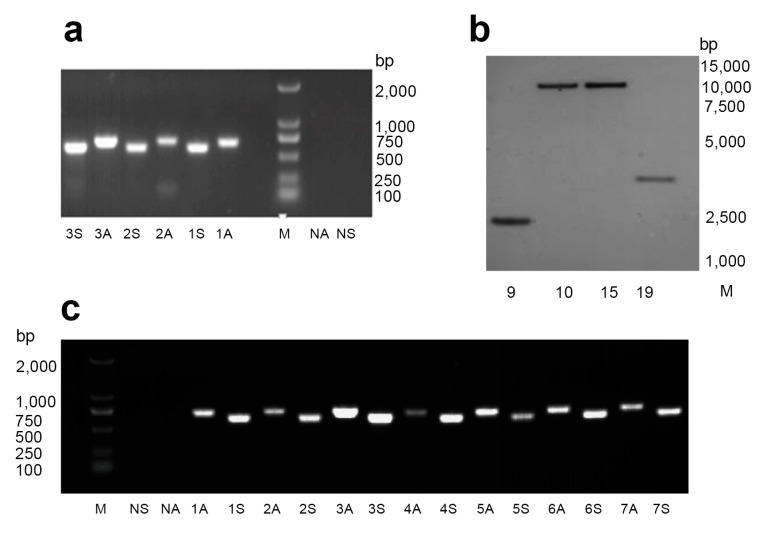
Molecular detection of transgenic cotton lines. (**a**) PCR analysis of cotton expressing *HaHR3* with primers amplifying sense and anti-sense fragments. Lanes 1S–3S denote PCR analysis of lines 5, 7, and 9 with sense fragment primers. Lanes 1A–3A denote PCR analysis of lines 5, 7, and 9 with anti-sense primers. Lanes NA and NS are antisense and sense negative controls, respectively. M: DL2000 Marker; (**b**) southern blot analysis of transgenic cotton. 9, 10, 15, and 19 denote transgenic cotton lines. M: DL15000 Marker; (**c**) RT-PCR analysis of cotton expressing *HaHR3* with primers amplifying sense and anti-sense fragments. Lanes 1S–7S denote PCR analysis of lines 5, 7, 8, 9, 10, 15, and 19 with sense fragment primers. Lanes 1A–7A denote PCR analysis of lines 5, 7, 8, 9, 10, 15, and 19 with anti-sense primers. Lanes NA and NS are antisense and sense negative controls, respectively. M: DL2000 Marker.

Sense and antisense fragments of the 19 lines were amplified by PCR and showed positive signals of *HaHR3* (Figure 1a). Southern blotting identified 12 single-copy transgenic lines (Figure 1b). Based on *HaHR3* expression levels in transgenic cotton plants determined by RT-PCR (Figure 1c), seven positive single-copy cotton lines (lines 5, 7, 8, 9, 10, 15, and 19) were selected and used for further investigation.

### 2.2. HaHR3 Transgenic Cotton Showed Enhanced Protection from Bollworms

**Figure 2:**
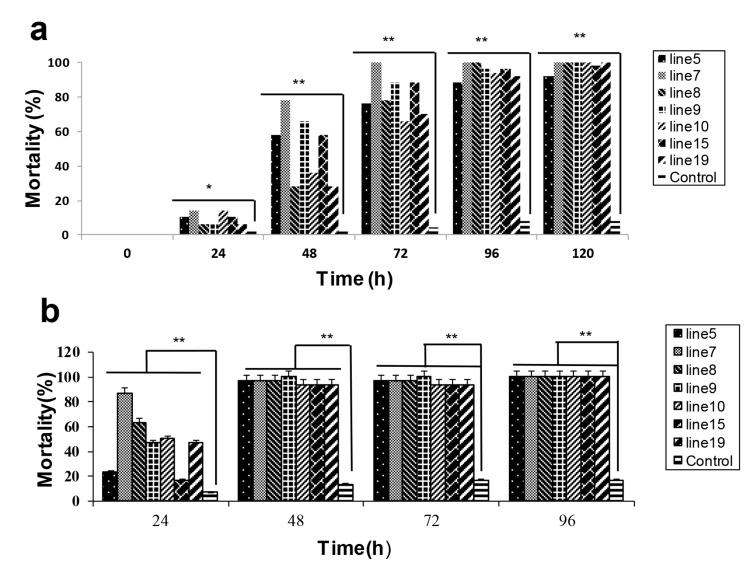
*H. armigera* mortality of newly hatched and 3rd-instar larvae after feeding on transgenic cotton. Mortality of newly hatched (**a**) and 3rd-instar larvae (**b**) after feeding on different lines of transgenic cotton (lines 5, 7, 8, 9, 10, 15, and 19). Male parent F15 cotton was used as a control. Error bars represent means ± SD. Essentially identical results were obtained across three independent experiments. Asterisks indicate significant differences from the control (* *p* < 0.05, ** *p* < 0.01).

After feeding on transgenic *HaHR3* cotton plants, the mortalities of newly hatched, 3rd-instar and 5th-instar larvae of *H. armigera* were significantly higher than the control group (Figure 2a). After 2 days of feeding, the mortalities of newly hatched larvae were 58%, 78%, 28%, 66%, 36%, 58%, and 28%, in groups from line 5, line 7, line 8, line 9, line 10, line 15, and line 19, respectively; control group mortality was 2%. After 5 days of feeding, larvae in the transgenic groups were almost all dead; however, the control group mortality was 8%. After feeding for 1 day, the mortalities of 3rd-instar larvae in each group were 23.33%, 86.67%, 63.33%, 46.67%, 50.00%, 16.67%, and 46.67%, respectively (Figure 2b). After 2 days of feeding, the mortalities were almost 100%, while the control group mortality was only 16.67% after 4 days of feeding. Additionally, the feeding amounts of newly hatched and 3rd-instar larvae in transgenic cotton groups were much lower than those in the control groups.

**Figure 3:**
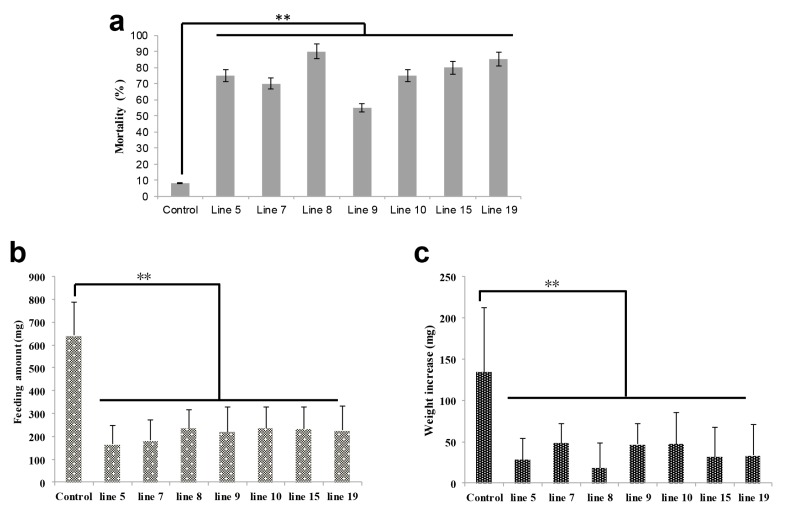
*H. armigera* mortality, feeding amount, and weight increase of 5th-instar larvae after feeding on transgenic cotton. Mortality (**a**), feeding amount (**b**), and weight increase (**c**) of 5th-instar larvae after feeding on different lines of transgenic cotton (lines 5, 7, 8, 9, 10, 15, and 19) for 3 days. Male parent F15 cotton was used as a control. Error bars represent mean ± SD. Essentially identical results were obtained across three independent experiments. Asterisks indicate significant differences from the control (** *p* < 0.01).

5th-instar larvae growing normally were fed on transgenic and control cottons, respectively. After 3 days of feeding, high mortality was observed in all 5th-instar larvae fed the seven transgenic lines (Figure 3a). At this time, the surviving 5th-instar larvae in the transgenic groups and the control group had average feeding amounts of 214.67 mg and 642.64 mg, respectively, and average weight increases of 36.54 mg and 134.28 mg, respectively (Figure 3b,c).

### 2.3. HaHR3 Transgenic Cotton Affected the Development and Propagation of H. armigera

**Figure 4:**
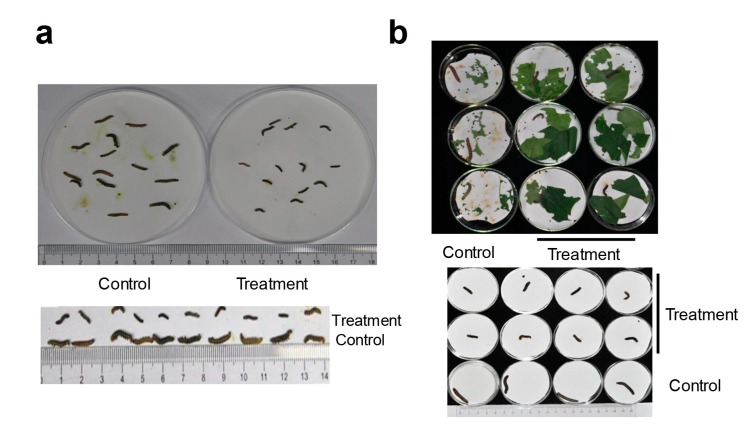
Deformed development of *H. armigera* at the metamorphosis stage. (**a**) Phenotype of the 3rd-instar larvae; (**b**) phenotype of the 5th-instar larvae. The control group was fed with male parent F15 cotton; treatment groups were fed with transgenic cotton.

**Figure 5:**
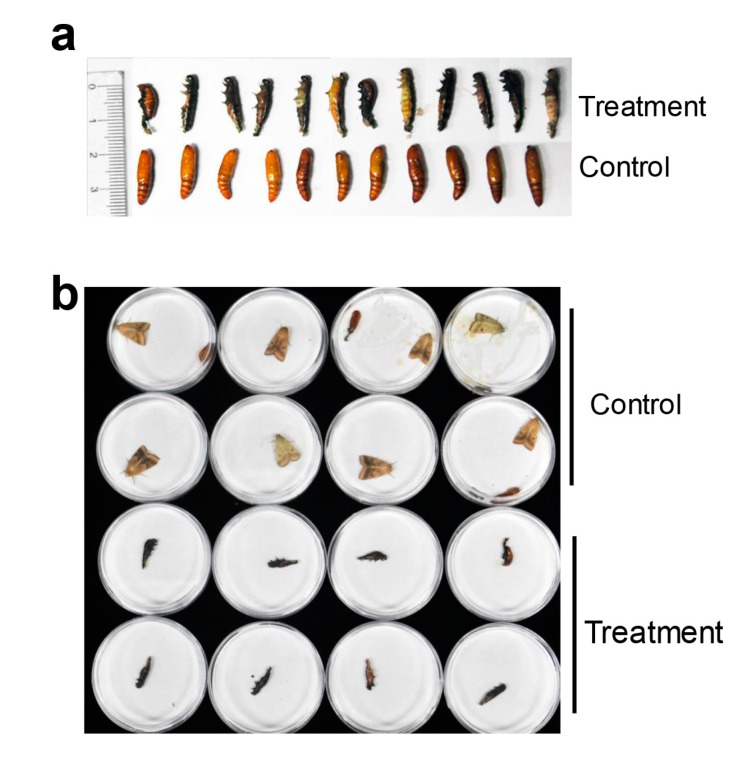
Developmental deformity phenotype of *H. armigera* at the pupal and emergence stage. (**a**) The inhibition of pupation of 5th-instar larvae after feeding with transgenic cotton expressing *HaHR3*; (**b**) the deformity of adult emergence. The control group was fed with male parent F15 cotton; treatment groups were fed with transgenic cotton.

When feeding on *HaHR3* transgenic cotton, deformed bollworm development was observed at the metamorphosis stage. After feeding for three days, 3rd-instar larvae exhibited abnormal phenotypes. Treated bollworms were smaller than the controls, and some bollworms were weaker and moved slowly or not at all (Figure 4a). 5th-instar larvae in the transgenic groups refused to eat, and treated larvae were smaller than controls (Figure 4b). Many bollworms failed to complete the molting process. The surviving 5th-instar larvae showed a high rate of pupation deformity (Table 1; Figure 5a). Additionally, some new puparia were not integrated into the pupal body (Figure 5b), and the resulting adults were deformed.

### 2.4. HaHR3 Expression in H. armigera after Feeding on Transgenic Cotton Plants

**Figure 6:**
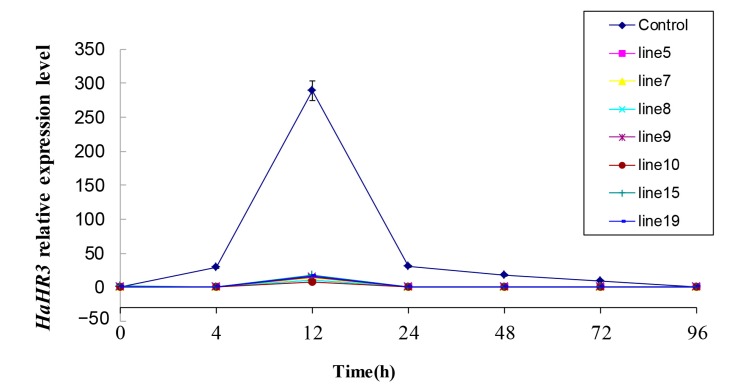
The relative expression of *HaHR3* in *H. armigera*. qRT-PCR analysis of *HaHR3* expression. *HaHR3* relative expression levels in *H. armigera* fed on transgenic cotton (lines 5, 7, 8, 9, 10, 15, and 19) expressing *HaHR3*. Male parent F15 cotton was used as a control. Larvae were in 3rd-instar at 0 h. Error bars represent mean ± SD. Essentially identical results were obtained across three independent experiments.

*HaHR3* expression in larvae was examined by qRT-PCR at different intervals (Figure 6), and was shown to have a periodic expression profile of 12 h. The result of qRT-PCR showed that *HaHR3* was expressed in all seven positive lines and control cotton, while the transcription levels of dsRNAs in these lines appeared diversely. *HaHR3* expression showed a 30-fold increase in *H. armigera* feeding on control cotton as compared with those feeding on transgenic cotton at 12 h. After 12 h, the *HaHR3* expression in *H. armigera* feeding on control cotton was also higher than that feeding on transgenic cotton until larvae completing molt at 96 h. *HaHR3* expression could be suppressed by all seven transgenic cotton lines expressing *dsHaHR3*, and there was no significant difference in suppression levels among the groups.

### 2.5. HaHR3 Transgenic Cotton Showed an Improved Cotton Yield

A wide range of traits was observed among control and transgenic cotton lines, and there was no significant difference between poll weight, lint percentage (%), upper half mean fiber length (mm), and bundle strength (cN/tex). However, poll number, micronaire, and uniformity index of the seven transgenic cotton lines were higher than those of the control (Table 2). Compared with the control F15, the transgenic cotton boll shape varied from cone to ovoid, transgenic boll shells were much thinner, and the open boll was much smoother. Thus, the transgenic cotton lines expressing *HaHR3* showed an improved cotton yield when compared with the control.

## 3. Discussion

RNAi is typically induced in insects by the microinjection or feeding of dsRNAs to silence target genes. RNAi by feeding has several distinct advantages over injection, including its reduced labor intensity and cost, the ability to be performed on large numbers of genes, and its durability [25]. Plant-mediated RNAi has emerged as an effective approach to protect plants against insect pests. In the present work, cotton was successfully transformed with pCAMBIA-RNAi-*HaHR3* to obtain 7 positive transgenic cotton lines that were then fed to *H. armigera*.

HR3 participates in the regulation of molting and metamorphosis. HHR3 is a molt-regulating transcription factor in *H. armigera*, and its different expression patterns exhibit variable effects on the molting, feeding, and development of different instar larvae [22]. CHR3, a critical transcriptional regulator in *Caenorhabditis elegans*, plays an important role in molting [26], and DHR3 is required for the development of adult bristles, wings, and cuticles, and prepuparium formation in *Drosophila* metamorphosis [27].

A previous study designed four different fragments covering the full CDS of *HaHR3*, and their independent transformation in *Escherichia coli* had different effects on the control of *H. armigera* [24]. The 5′-end 450 bp of dsRNA-*HaHR3* as expressed by tobacco resulted in the highest level of mortality and developmental deformity in *H. armigera* metamorphosis. In the present study, this fragment was also selected for expression in transgenic cotton for plant mediated-RNAi research. The mortalities and developmental deformities of newly hatched, and 3rd- and 5th-instar larvae fed transgenic cotton groups expressing *HaHR3* were significantly higher than the control group. This suggested that *HaHR3* feeding could affect the normal development of bollworms or even kill them at different instar stages. Therefore, *HaHR3* transgenic cotton could enhance the protection against bollworms. Moreover, the observed abnormal pupation and eclosion indicated that *HaHR3* transgenic cotton further affects the propagation of *H. armigera*, which is in agreement with previous findings [24].

*HaHR3* transgenic cotton is more efficient at silencing the molt-regulating transcription factor gene of *H. armigera* than *HaHR3* transgenic tobacco. *HaHR3* was successfully silenced after 12 h of feeding *H. armigera* with transgenic cotton leaves as compared with after 60 h when feeding with transgenic tobacco leaves expressing *HaHR3* [24]. Additionally, all seven transgenic cotton lines showed a higher mortality and pupation deformity when compared with transgenic tobacco. *HaHR3* transgenic cotton may also prevent damage from bollworms and affect their future development.

Transgenic cotton plants expressing the P450 genes *CYP6AE14* and *CYP6B6* acquired enhanced resistance to cotton bollworms by plant-mediated RNAi [28,29]. *HaHR3* transgenic cotton produced similar adverse effects on larval growth to *CYP6AE14* transgenic cotton, and caused more obvious growth retardation than *dsCYP6AE14* transgenic cotton [29]. Moreover, *HaHR3* transgenic cotton resulted in a significantly higher larval mortality rate than *CYP6B6* transgenic cotton. In addition, transgenic cotton expressing dsRNA of a *HMGR* gene showed similar growth, development, mortalitis, and pupation of cotton bollworms [30]. The process of selecting a target gene for silence is of paramount importance [31]. *HaHR3* is a hormone receptor, and *HMGR* is a reductase of juvenile hormone. Both of them related to molting process, while P450 induced by gossypol. These results indicate that different kinds of target genes have varying silencing effects on cotton bollworms.

The present study showed that transgenic cotton expressing *HaHR3* improved cotton yield in that the cotton boll number, shape, shell, and open boll were superior to those of control F15. These results indicated that no loss of fitness in cotton yield. The uniformity index and micronaire of the seven transgenic cotton lines were also slightly improved as compared with the control. Micronaire measures the fiber fineness and maturity, and values of 3.5–4.9 are optimal when compared with values <3.4 or >5.0 [32]. A moderate micronaire and better uniformity index could be beneficial in producing high-quality yarns [33]. However, there was no obvious difference in fiber quality among the control and transgenic cotton lines.

## 4. Materials and Methods

### 4.1. Plant Expression Vector Construction and Cotton Plant Transformation

*HaHR3* (GenBank accession number: FJ009448), containing a 1671 bp full coding sequence (CDS), was cloned from *H. armigera* in a previous study [23]. Multiple sequence alignment showed that *HaHR3* shares 98.50% identity with *HHR3* as cloned by Zhao [22]. The 5′ terminal 450 bp of dsRNA-*HaHR3* was selected for plant mediated-RNAi research. Sense fragment of dsRNA-*HaHR3* was digested with *Xho*I and *Bgl*II, and anti-sense fragment was digested with *Sal*I and *Bam*HI. The primers used are listed in Table 3. Sense and anti-sense fragments of dsRNA-*HaHR3* were cloned into the intermediate vector pRNAi1017. Then, double-digested pRNAi1017 and plant over-expression vector pCAMBIA2300-35s-OCS with *Sal*I and *Pst*I. pCAMBIA2300-35s-OCS, and target gene fragments were ligated with introns using TOYOBO Ligation high Ver. 2 (Japan, lot 94190H6, codeLGK-101) [23]. Recombinant plasmid pCAMBIA-RNAi-*HaHR3* was used to pollen tube pathway-mediated cotton transformation.

Xiangza cotton male parent F15 was grown until flowering at the breeding base in Hainan Province. Pollen tube pathway-mediated cotton transformation was conducted according to a previous study [34]. Each flower was injected with 10 µL plasmid DNA (100 µg/mL) containing dsRNA-*HaHR3* using a 50 µL microinjector. The concentration of gibberellic acid used was 50 mg/L.

Transgenic cotton seeds were harvested and sown at the breeding base of the Cotton Science Research Institute of Hunan Province in Changde. The first resistance selection to kanamycin (2500 mg/L) was conducted at the two-leaf stage of transgenic cotton. Cotton plants showing spotted leaves were pulled up. Three kanamycin applications (2500, 3000, and 4000 mg/L) were then made to select for resistance, and cotton plants positive for kanamycin resistance were marked and cultivated for harvesting seeds.

### 4.2. Insect and Plant Culture

Cotton bollworm eggs were purchased from Baiyun Industry Co., Ltd. (Jiyuan, China). Larvae were fed an artificial diet as previously described [35], and reared in a versatile environmental test chamber (SANYO, MLR-350HT) at 28 °C with 70% relative humidity under a 14-h light/10-h dark photoperiod. Larvae were fed individually in 24-well plates. Cotton plants were grown in a greenhouse for further research.

### 4.3. Transgenic Cotton Molecular Analysis

Genomic DNA was isolated from the leaves of *HaHR3* cotton seedlings using the cetyltrimethylammonium bromide method [36]. *HaHR3* primer pairs were designed to amplify sense and antisense fragments from each transgenic line (Table 1). Genomic DNA was digested using *Bam H*Ι and *Eco R*Ι, separated via 1.2% agarose gel electrophoresis, and then transferred onto Hybond-N nylon membranes (Amersham, Uppsala, Sweden) for Southern blotting [37]. The ds*HaHR3* probe used in the experiment was labeled using the DIG-High primer method, and hybridization was performed with the DIG-High Primer DNA Labeling and Detection Starter Kit I (Roche, Germany). Total RNA was isolated from each transgenic line using TRIzol reagent (Invitrogen, Carlsbad, CA), according to the manufacturer’s instructions. First-strand cDNA was synthesized using reverse transcriptase II (Transgen, Beijing, China) under the following reaction conditions: 37 °C for 10 min, 42 °C for 1 h, and 95 °C for 5 min. Primers were designed to amplify the CDS of the *HaHR3* fragments (Table 3).

### 4.4. Quantitative Real-Time (qRT)-PCR Analysis

Total RNA of bollworms was extracted from experimental and control cotton bollworms using TRIzol reagent. The FastQuant RT Kit (Tiangen, Beijing, China) was used to synthesize first-strand cDNA under the following conditions: 95 °C for 3 min, then 27 cycles of 94 °C for 30 s, 62 °C for 30 s, and 72 °C for 45 s, followed by 72 °C for 10 min. For qRT-PCR, SYBR Green (Tiangen, Beijing, China) was used with a CFX96 real-time PCR machine (Bio-Rad Laboratories, Hercules, CA, USA). Thermal cycling conditions were as follows: denaturation at 95 °C for 15 s, followed by 40 cycles of 95 °C for 10 s, and 60 °C for 30 s. Actin (GenBank accession no. X97615) was amplified as an internal control. Each reaction was performed in triplicate. qRT-PCR primers are listed in Table 3.

### 4.5. Feeding Bioassays with Cotton Expressing HaHR3

To investigate the transgenic cotton activity, seven independent single-copy lines were chosen from 19 transgenic plants for the *HaHR3* feeding bioassay. Male parent F15 cotton was used as the control. A total of 50 larvae from each group were used, and each group contained three replicates. Newly hatched, 3rd-instar, and 5th-instar synchronous larvae were selected and fed individually with cotton leaf discs of the same size in 6-well plates. The leaf disc was replaced every 12 h. Wet filter paper of the same size was placed in every chamber to maintain humidity.

To calculate the feeding amount and weight increase, each fresh cotton leaf disc and 5th-instar larva was weighed individually. After feeding on different diets for indicated times, each cotton leaf disc and r larva was weighed individually again. The survival larvae from each treatment were kept and recorded for further pupation or adult emergence deformity rate calculations.

### 4.6. Cotton Yield and Fiber Quality Measurement

Transgenic cotton lines were collected during the harvesting period for yield analysis at the breeding base. Boll number, boll weight (g), and lint (%) were measured. To test the fiber quality of transgenic cotton lines and F15 cotton, the following measurements were included: upper half mean fiber length (mm), bundle strength (cN/tex), micronaire, and uniformity index (%). Lint samples (15 g) of each line were analyzed using USTER HVI 1000 (USTER Technologies, Inc., Uster, Switzerland). The fiber microstructure detection and cellulose test were performed according to a previous study [38].

### 4.7. Statistical Analysis

All experiments were repeated three times. Statistical analysis was carried out using the Statistical Analyses System for Windows v8 (SAS, Cary, NC, USA). Data are presented as means ± standard deviation. Variance analysis was conducted by comparing statistical differences based on the Student’s *t*-test (*p* < 0.05).

## 5. Conclusions

In summary, feeding *H. armigera* transgenic cotton expressing *HaHRs* can affect the feeding amount, weight increase, molting, pupation, and eclosion of bollworms, as well as causing abnormal phenotypes or death. This technique therefore enables insect pests to be controlled in the field. As a recently developed genetic modification, the technology of plant-mediated RNAi could lead to a broad application in the protection of crops against insects.

## Figures and Tables

**Table 1:** The pupation deformity and eclosion rate of *H. armigera* development.

Line	Pupation Deformity Rate/%	Eclosion Rate/%
Control	0	100
Line 5	100	0
Line 7	83.3	16.7
Line 8	100	0
Line 9	78.8	21.2
Line 10	80	20
Line 15	100	0
Line 19	100	0

**Table 2:** Cotton yield and fiber quality of transgenic cotton expressing *HaHR3*.

Line	Boll Number	Poll Weight (g)	Lint (%)	Fiber Quality			
				Upper-Half Mean Fiber Length (mm)	Bundle Strength (cN/tex)	Micronaire	Uniformity Index
F15	18.5 ± 2.6 A	6.3 ± 0.30 A	41.5 ± 0.6 A	31.13 ± 0.32 A	30.7 ± 0.8 A	5.34 ± 0.15 A	145 ± 1.5 A
Line5	29.5 ± 2.7 B	5.8 ± 0.54 A	41.2 ± 0.4 A	31.43 ± 0.63 A	31.4 ± 0.92 A	4.64 ± 0.56 B	150 ± 2.6 B
Line7	28.4 ± 3.6 B	6.2 ± 0.15 A	38.9 ± 0.7 A	28.79 ± 0.65 A	32.4 ± 1.22 A	4.65 ± 0.43 B	147 ± 2.8 A
Line8	26.5 ± 3.2 B	5.9 ± 0.24 A	39.8 ± 0.9 A	31.22 ± 0.82 A	31.5 ± 0.72 A	4.96 ± 0.42 B	158 ± 3.8 B
Line9	22.4 ± 2.2 B	6.3 ± 0.22 A	40.3 ± 0.7 A	30.45 ± 0.26 A	30.8 ± 0.76 A	4.87 ± 0.88 B	149 ± 2.3 B
Line10	26.8 ± 3.4 B	6.1 ± 0.65 A	39.5 ± 0.5 A	30.12 ± 0.23 A	31.8 ± 1.21 A	4.92 ± 0.28 B	157 ± 2.4 B
Line15	34.2 ± 3.4 B	5.8 ± 0.18 A	39.7 ± 0.2 A	30.13 ± 0.44 A	32.6 ± 0.95 A	5.25 ± 0.24 A	144 ± 3.2 A
Line19	30.5 ± 2.6 B	6.0 ± 0.28 A	39.5 ± 0.7 A	30.14 ± 0.64 A	33.4 ± 1.12 A	4.74 ± 0.16 B	168 ± 1.9 B

A/B: Different letters indicate significance at *p* < 0.01.

**Table 3:** Sequences of primers used in this study.

Target Gene	Accession No.	Primer Sequence
*HaHR3*	FJ009448	F:5′-*cc*CTCGAGATGAACAACAACCAGTTCCACGAT-3′
		R:5′-*ga*AGATCTCACGCAGGCTTTATTCCGTGGACA-3′
Sense-Frag.	FJ009448	F:5′-*cc*CTCGAGATGAACAACAACCAGTTCCACGAT-3′
		R:5′-*ga*AGATCTCACGCAGGCTTTATTCCGTGGACA-3′
Anti-sense Frag.	FJ009448	F:5′-*gc*GTCGACATGAACAACAACCAGTTCCACGAT-3′
		R:5′-*cg*GGATCCCACGCAGGCTTTATTCCGTGGACA-3′
qRT-*HaHR3*	FJ009448	F:5′-GCCACAGGATGTCTCCAAGC-3′
		R:5′-GAGCCAGATTTCAAGAGCAAAA-3′
qRT-*actA3b*	X97615	F:5′-CCCCGTCCACAATGAAGATC-3′
		R:5′-GGCCAGACTCGTCGTACTCCT-3′

Restriction endonuclease cleavage sites CTCGAG (XhoI), AGATCT (BglII), GTCGAC (SalI) and GGATCC (BamHI) are underlined.
